# From Unexplained Iron-Deficiency Anaemia to Ileo-Ileal Intussusception: A Case Report of Primary Small Bowel Leiomyosarcoma

**DOI:** 10.7759/cureus.116419

**Published:** 2026-09-17

**Authors:** Manas M Potdar, Amanda Barden, Kopinath Kathirvetpillai, Anang Pangeni, Ashish Shrestha

**Affiliations:** 1 First Faculty of Medicine, Charles University, Prague, CZE; 2 Department of General Surgery, William Harvey Hospital, Ashford, GBR; 3 Department of Pathology, William Harvey Hospital, Ashford, GBR

**Keywords:** adult intussusception etiology, adult small bowel intussusception, emergency laparoscopic surgery, histopathology of leiomyosarcoma, ileo-ileal intussusception, immunohistochemistry of leiomyosarcoma, intussusception diagnosis and treatment, iron-deficiency anaemia (ida), small bowel leiomyosarcoma, smooth muscle malignancy

## Abstract

Intussusception refers to the telescoping of a gastrointestinal segment into an adjacent distal segment. Adult intussusception is rare and is often associated with a lead point (LP). Primary small bowel leiomyosarcoma (LMS) is a malignancy of smooth muscle cells that arises from the bowel wall and can serve as an LP for intussusception. We report the case of a man in his 60s with long-standing unexplained iron-deficiency anaemia (IDA) who underwent multiple upper and lower gastrointestinal endoscopic investigations, but the source of the anaemia could not be identified. Years after the initial diagnosis of IDA, he presented with abdominal pain, nausea, vomiting, constipation, and an unintentional weight loss of 6.4 kg over a 10-day period. Computed tomography of the abdomen and pelvis demonstrated ileo-ileal intussusception. Emergency laparoscopic-assisted segmental small bowel resection with side-to-side anastomosis was performed. Histopathological examination demonstrated a spindle-cell malignancy with mucosal ulceration. The ulcerated intraluminal tumour represented a plausible source of chronic occult gastrointestinal blood loss and subsequently served as the LP for intussusception. Immunohistochemistry confirmed small bowel LMS and excluded gastrointestinal stromal tumour. His postoperative recovery was uneventful, and he was discharged three days after surgery. At the two-month follow-up, his anaemia had resolved without perioperative blood transfusion, although mild residual iron deficiency remained.

## Introduction

Intussusception refers to the telescoping of a gastrointestinal segment into an adjacent distal segment [1]. Adult intussusception is rare and is often associated with an underlying pathological lead point (LP). An LP is a lesion within the intestinal wall that is drawn distally by peristalsis along with the proximal bowel segment, resulting in intussusception. Neoplasms, either benign or malignant, account for 60% of identified LPs [2]. Ileo-ileal intussusception is a specific subtype of entero-enteric intussusception [3]. Clinical presentation in adults includes nonspecific symptoms such as abdominal pain, nausea, and vomiting [2]. If left untreated, intussusception can lead to necrosis, peritonitis, sepsis, and perforation [2].

Leiomyosarcoma (LMS) is a malignant soft-tissue neoplasm of smooth muscle differentiation that may arise in various organ systems, including the gastrointestinal tract. Small bowel LMSs are rare, accounting for approximately 0.1% to 3% of small bowel malignancies [4]. Patients with small bowel LMS may present with chronic abdominal pain, weight loss, and gastrointestinal bleeding [4].

We report the case of a man in his 60s with long-standing unexplained iron-deficiency anaemia (IDA) who underwent multiple upper and lower gastrointestinal endoscopic investigations, but the source of the anaemia could not be identified. Years after the initial diagnosis of IDA, he presented with ileo-ileal intussusception secondary to a primary small bowel LMS. The combination of histology and immunohistochemistry helped distinguish the LMS from a gastrointestinal stromal tumour (GIST). The intraluminal tumour showed mucosal ulceration, providing a plausible source of chronic occult gastrointestinal blood loss. It subsequently served as the LP for intussusception.

This case adds to the limited literature on small bowel LMS presenting with ileo-ileal intussusception and highlights the combination of long-standing unexplained IDA, unrevealing conventional endoscopic investigations and subsequent presentation with ileo-ileal intussusception requiring emergency surgery.

This case was previously presented as e-posters at the 8th Annual East Kent Surgical Conference on November 14, 2025 and the Association of Surgeons of Great Britain and Ireland International Surgical Congress 2026 on May 13, 2026. It was also presented as a short oral presentation at the 8th Nepalese Doctors Association UK Resident Doctors Conference on June 6, 2026.

## Case presentation

A man in his 60s presented to his general practitioner with intermittent abdominal pain, nausea, constipation, vomiting, and an unintentional weight loss of 6.4 kg over a 10-day period. Given these symptoms, his general practitioner arranged computed tomography of the abdomen and pelvis (CTAP). Three days after undergoing the CTAP, he presented to the emergency department with similar symptoms and was referred to the general surgery team. Table 1 summarises the timeline of the patient’s clinical presentation.

**Table 1:** Timeline of case presentation.

Day	Event
Day 1	General practitioner visit
Day 4	CT scan of the abdomen and pelvis was performed
Day 7	Emergency department visit
Day 8	Surgical team reviewed the patient. After CT scan review, the patient was prepared for emergency surgery. Surgery was performed
Day 11	Uneventful recovery + discharge

On surgical assessment, he reported crampy abdominal pain on the background of a constant dull ache. He did not report any lower urinary tract symptoms. While he complained of constipation, he noted this was a common side effect of his iron supplementation. His relevant past medical history included IDA, which had been diagnosed years before this presentation. For this, he underwent repeated gastroscopies and colonoscopies, but no source for the anaemia was identified. His past surgical history consisted of a previous inguinal hernia repair. He had also been placed under active surveillance for prostate cancer. He reported an allergy to penicillin and had no history of smoking or alcohol use. His regular medications included mebeverine, ferrous sulphate, and laxatives.

On physical examination, his abdomen was soft and non-tender, with no palpable masses or signs of peritonitis. There was no lymphadenopathy in the neck or in the groin. The CTAP demonstrated ileo-ileal intussusception without an obvious LP. A ‘target sign’ (Figure 1) and ‘bowel-within-bowel’ appearance (Figure 2), two characteristic signs of intussusception, were noted. There was mild upstream dilatation of the ileal loops (Figure 3) in the pelvis measuring approximately 4 cm in calibre. Faecal loading was noted in large bowel loops. There was no evidence of pneumoperitoneum or ascites. There were no clinical or radiological findings suggestive of bowel ischaemia. These CTAP findings confirmed the diagnosis of ileo-ileal intussusception.

**Figure 1:**
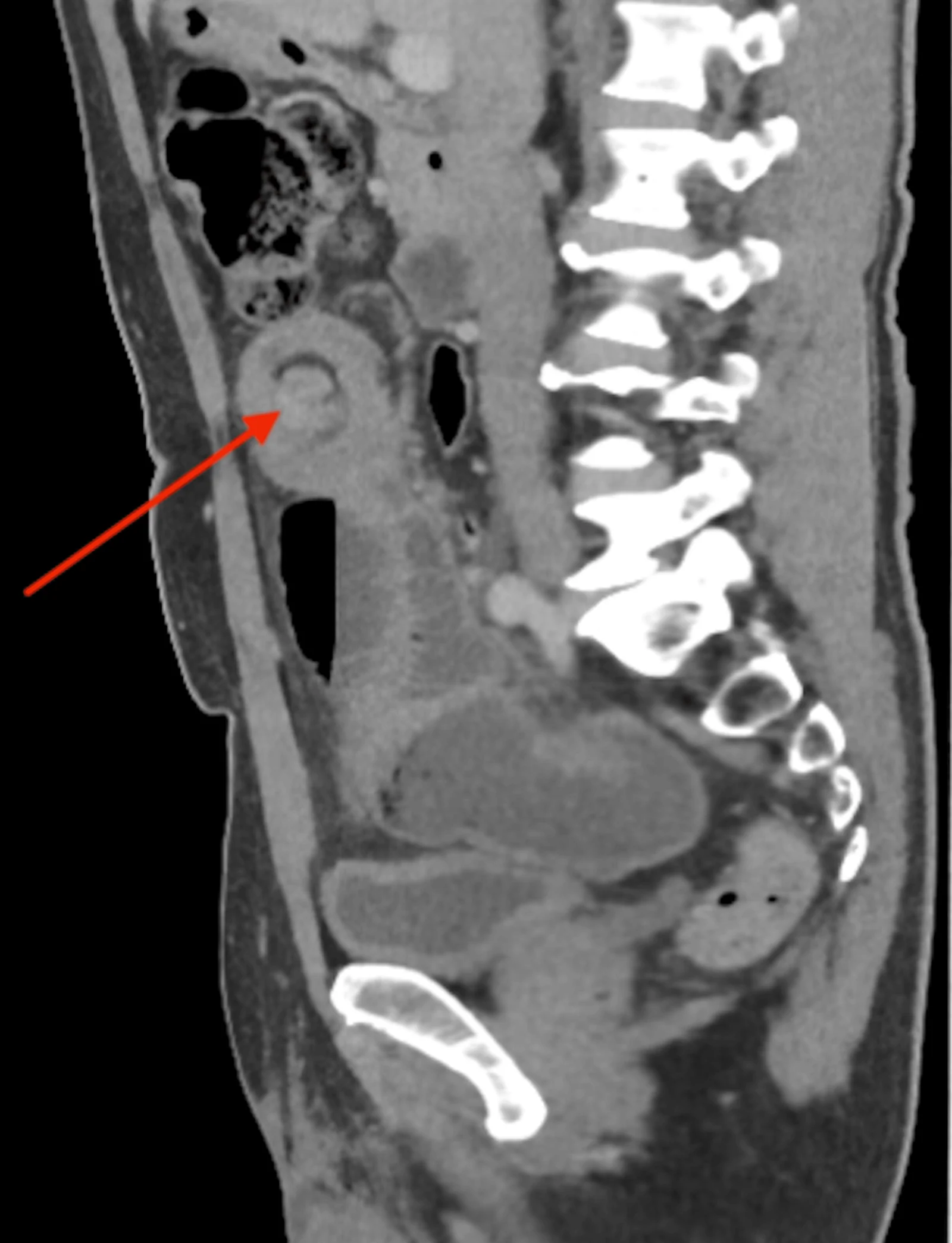
Sagittal section of CT of the abdomen and pelvis demonstrating the ‘target sign’ (red arrow).

**Figure 2:**
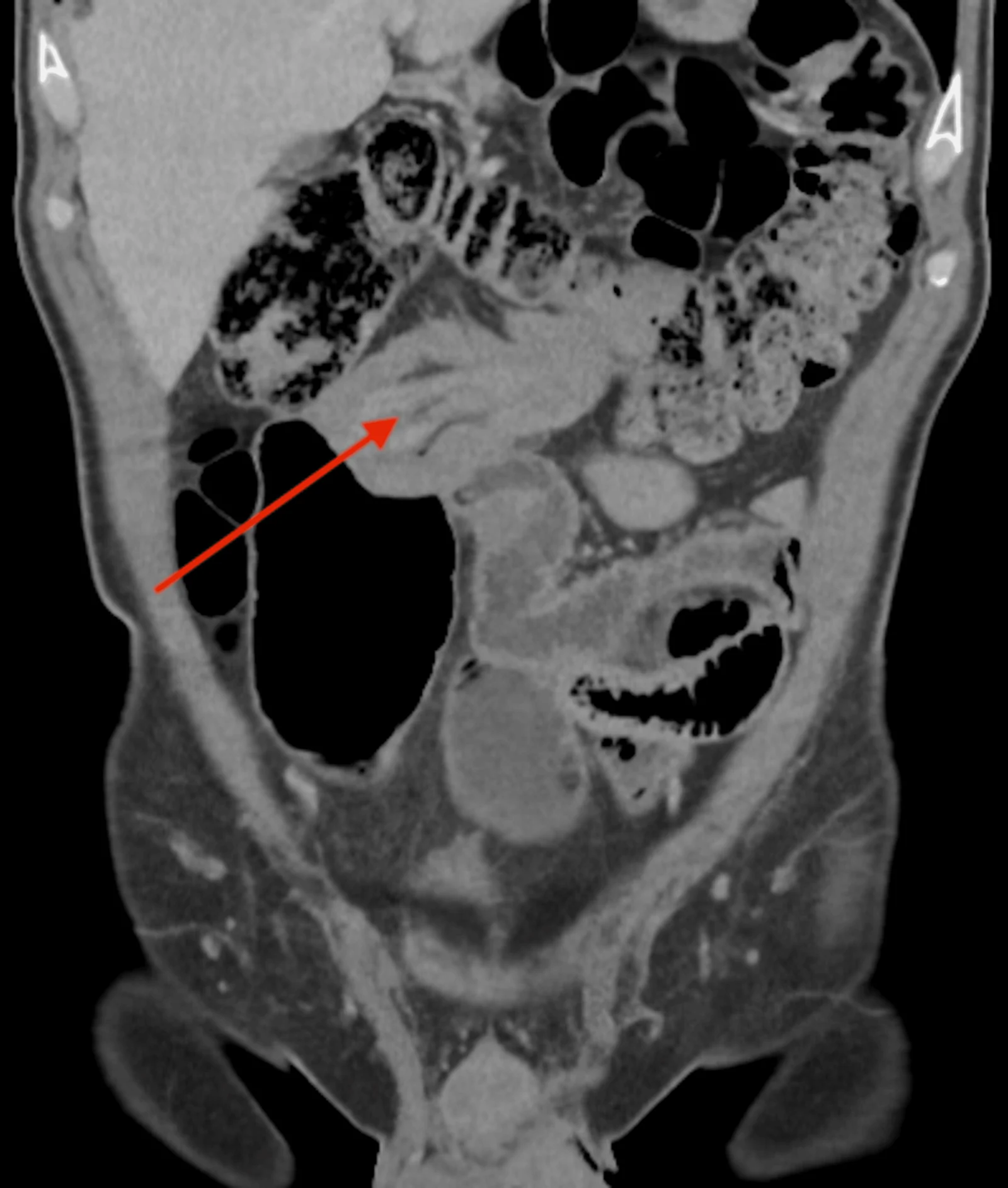
Coronal section of CT of the abdomen and pelvis showing the ‘bowel-within-bowel’ configuration (red arrow).

**Figure 3:**
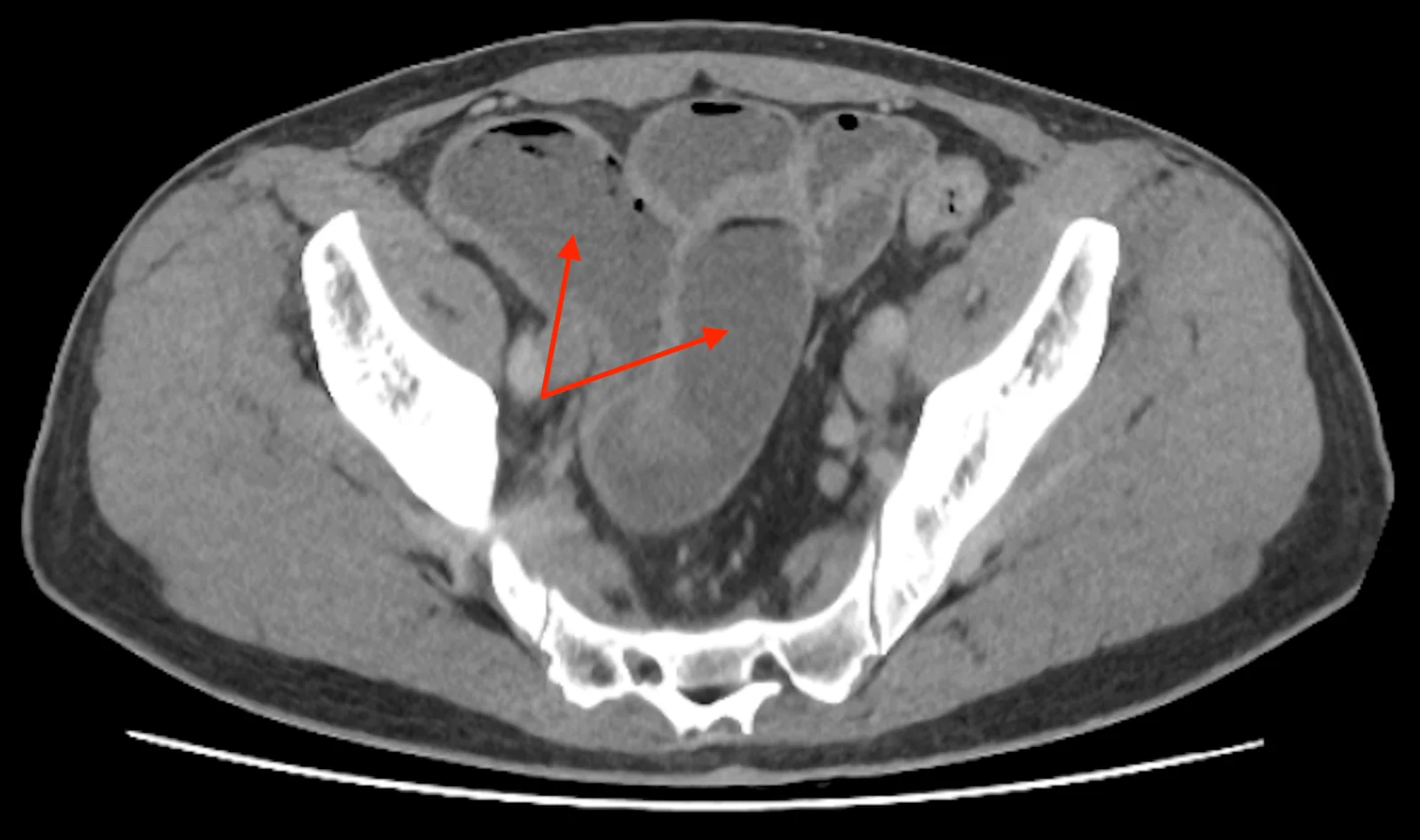
Transverse section of CT of the abdomen and pelvis demonstrating dilated proximal small bowel (red arrows).

Laboratory investigations reported a normal leukocyte count, an elevated C-reactive protein level of 60 mg/L, and a low haemoglobin level of 107 g/L. Iron studies performed one month before this emergency department presentation reported a serum iron level of 4.70 µmol/L, a transferrin level of 2.06 g/L, and a transferrin saturation of 9%.

His immediate supportive management included analgesia, intravenous fluids, ondansetron, prophylactic antibiotics (gentamicin and metronidazole), and venous thromboembolism prophylaxis (enoxaparin sodium). A nasogastric tube was inserted, and he was kept nil by mouth. Emergency surgery was planned because of the abdominal pain, CTAP findings of intussusception, and upstream small bowel dilatation consistent with small bowel obstruction.

Under general anaesthesia, the patient was placed in a modified Lloyd-Davies position. An infra-umbilical 10 mm port was inserted using the modified Hasson’s technique and a pneumoperitoneum was created. Two 5 mm working ports were placed. An ileo-ileal intussusception was identified in the right hypochondrium (Figure 4) together with proximal small bowel dilatation. The intussusception was exteriorised through a midline laparotomy with a wound protector. After partial reduction, a small bowel stricturing lesion was noted along with multiple prominent mesenteric lymph nodes, the largest measuring 15 mm. Oncological resection of the involved small bowel and mesentery was performed with appropriate macroscopic margins. The mesentery was cut and ligated, and the bowel was stapled with a 100 mm linear cutter. Isoperistaltic side-to-side anastomosis was performed in two layers. The mesenteric defect was closed, the bowel was reduced into the abdomen, and the abdominal cavity was irrigated with sterile water. The peritoneum, rectus sheath, and skin were closed, and a dressing was applied.

**Figure 4:**
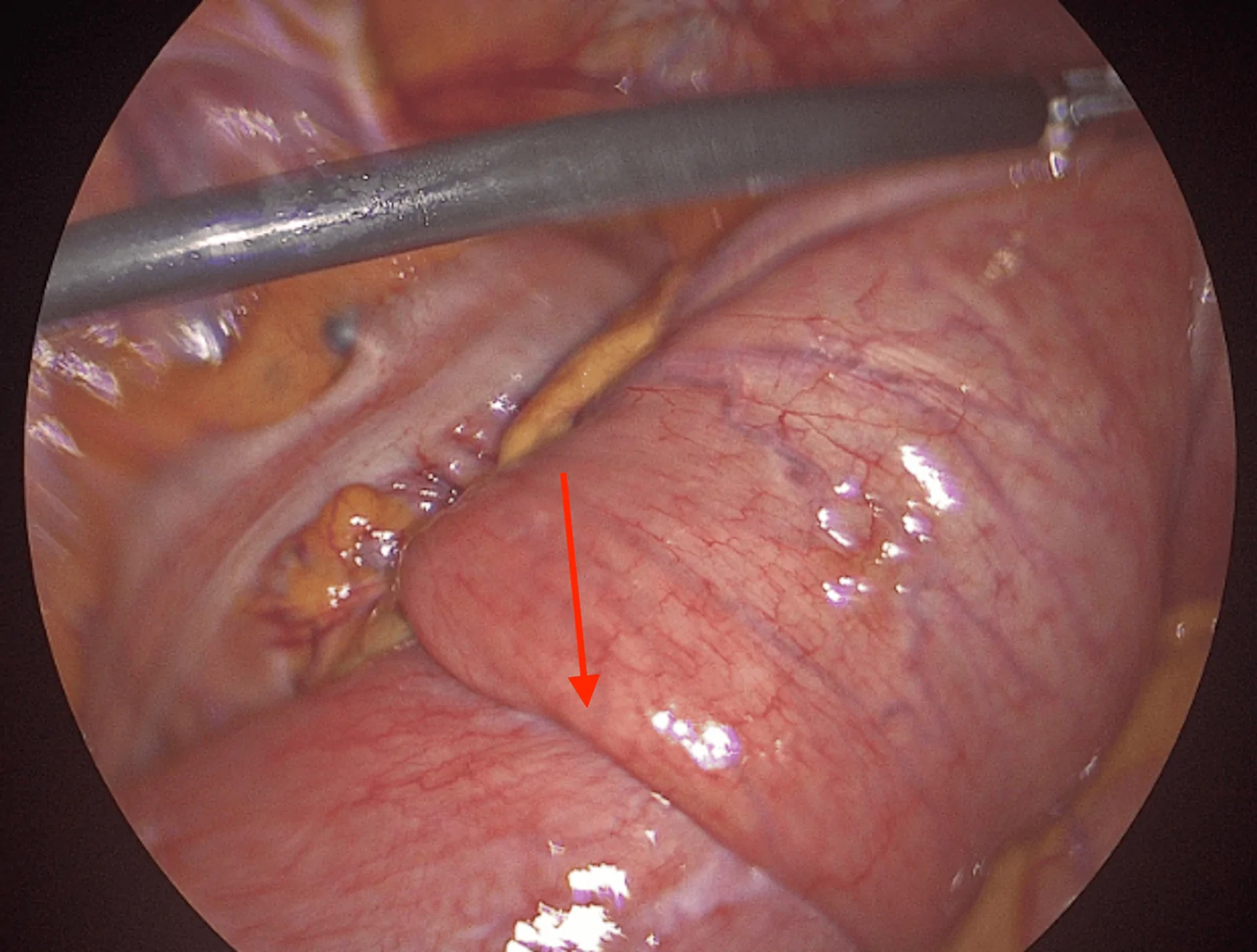
Intraoperative picture of the ileo-ileal intussusception (red arrow).

Postoperative care included intravenous fluids, enoxaparin sodium, catheter removal on postoperative day one, and appropriate mobilisation. He had an uneventful recovery and was discharged on postoperative day three.

The resected specimen was a 17 cm segment of ileum with attached mesentery. Within the lumen, a polypoid, rubbery, whitish tumour with an ulcerated mucosal surface (4.0 × 4.0 × 2.5 cm) was present (Figure 5). The mass was situated on the mesenteric border of the bowel and served as the LP of the intussusception. It caused marked luminal narrowing and proximal bowel dilatation. The serosal surface showed focal retraction but not full-thickness perforation. The tumour was well-demarcated from surrounding tissues. It was located 10 cm from the proximal resection margin, 4 cm from the distal margin, and at least 5.5 cm from the nearest mesenteric margin. The remaining adjacent mucosa showed no abnormality and all resection margins appeared grossly uninvolved.

**Figure 5:**
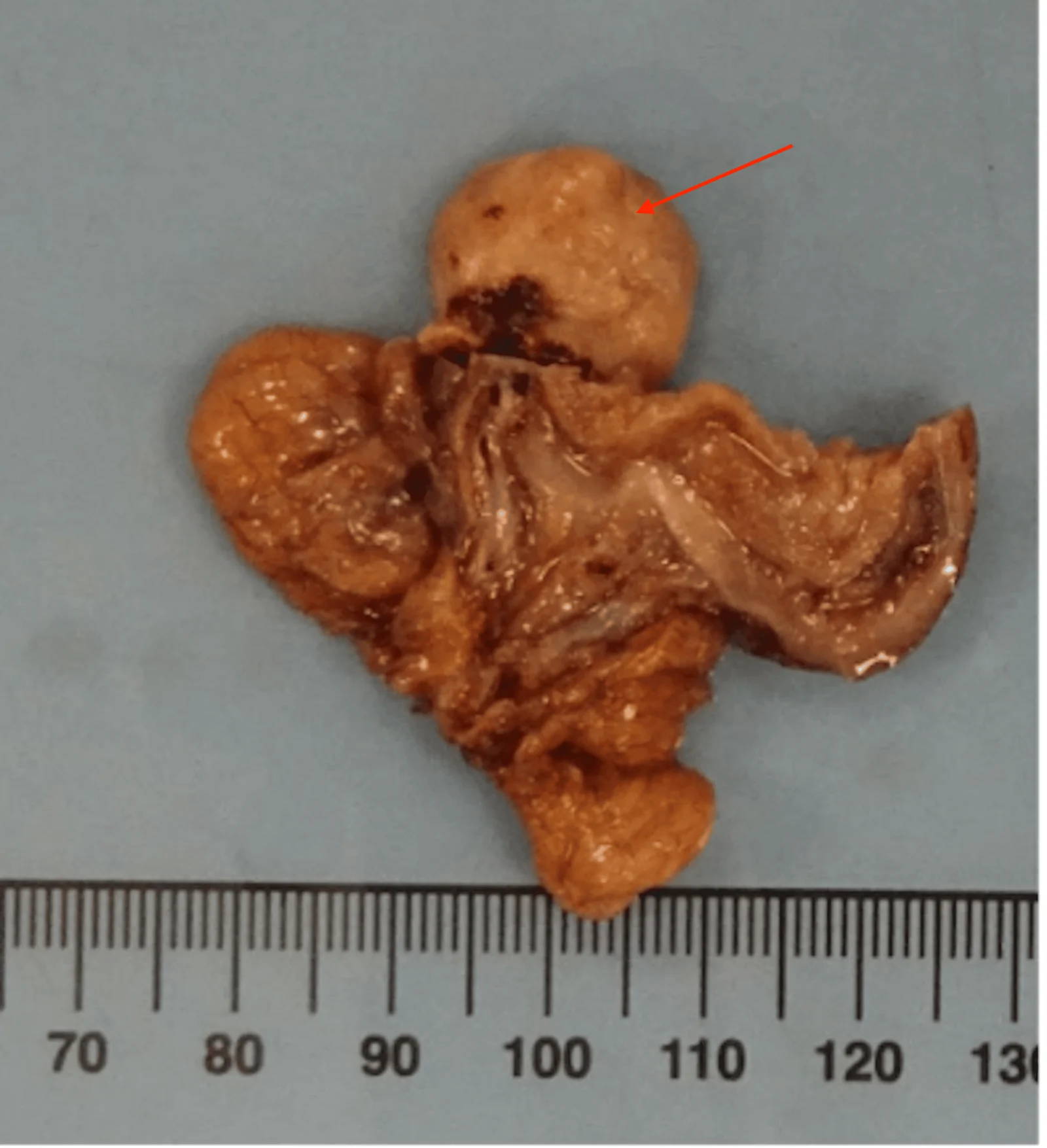
Gross appearance of the tumour with an ulcerated mucosal surface (red arrow).

Histological examination at low magnification revealed a malignant spindle cell neoplasm with an intersecting fascicular growth pattern within the bowel wall (Figures 6, 7). The neoplastic cells showed abundant eosinophilic cytoplasm and elongated, blunt-ended hyperchromatic nuclei, exhibiting marked nuclear pleomorphism (Figures 6, 7). High-power views highlighted cellular atypia and frequent mitotic figures, with an average of 15 per 10 high-power fields (Figure 8). No coagulative tumour necrosis was seen. The tumour invaded the submucosa and muscularis propria, but it did not penetrate the serosal surface. Mucosal ulceration was present. Lymphovascular invasion was not identified. Seven regional mesenteric lymph nodes showed reactive hyperplasia with no metastases. Both proximal and distal margins were free of tumour on microscopy.

**Figure 6:**
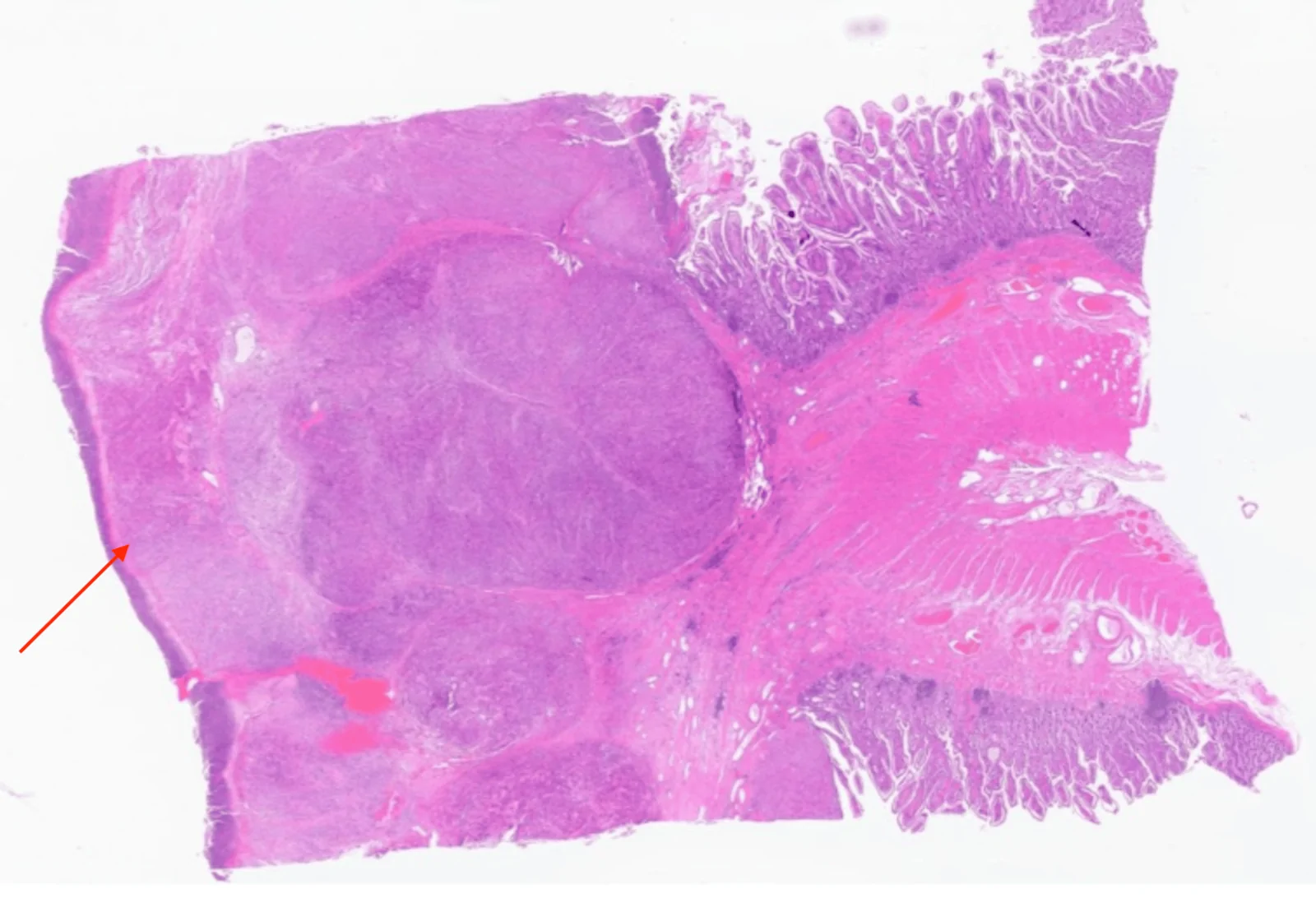
Scanning view showing a polypoid tumour with surface ulceration with a background of normal mucosa (red arrow).

**Figure 7:**
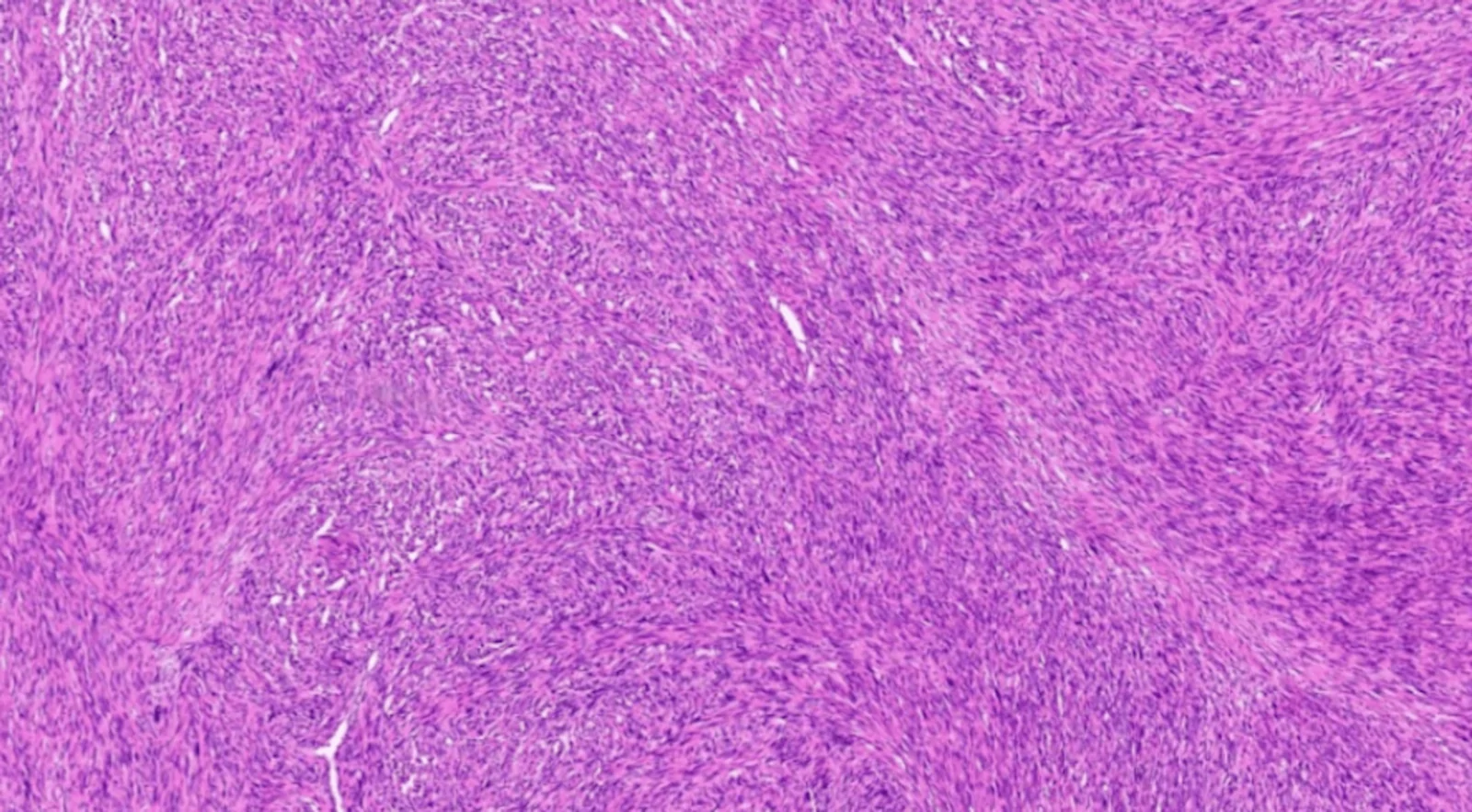
Low-power view (×5) showing neoplastic cells with abundant eosinophilic cytoplasm and elongated, blunt-ended hyperchromatic nuclei, demonstrating marked nuclear pleomorphism.

**Figure 8:**
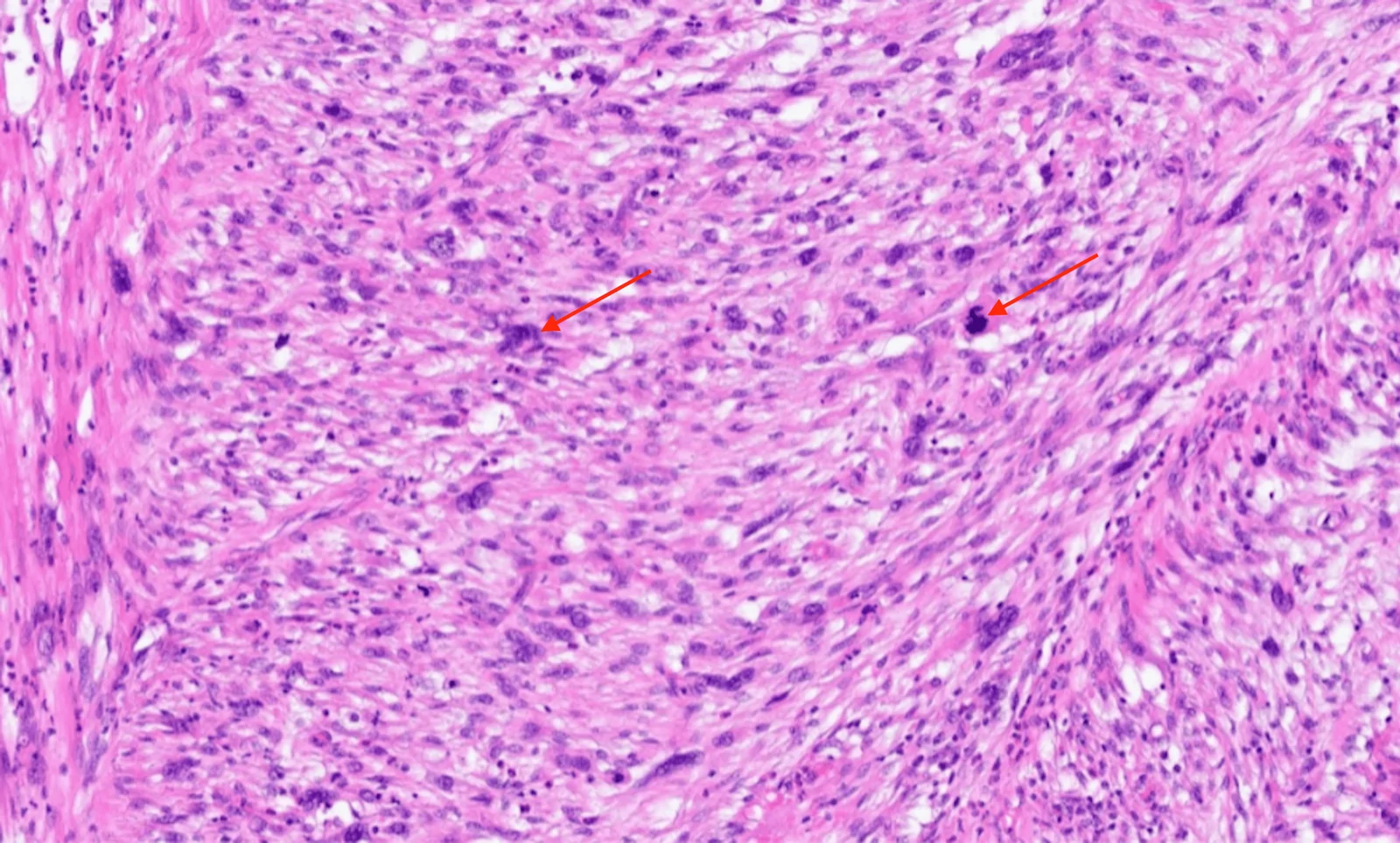
Histological slide showing pleomorphic nuclei and frequent mitotic figures at ×20 magnification (red arrows).

Immunohistochemistry confirmed smooth muscle differentiation of the neoplasm. The tumour cells showed diffuse strong positivity for smooth muscle actin (SMA) (Figure 9A) and desmin (Figure 9B). In contrast, no reactivity was seen for the epithelial marker AE1/3 (Figure 9C) or neural marker S-100 protein (Figure 9D). Importantly, markers associated with GIST were negative, with no expression of CD117/c-KIT (Figure 9E) or CD34 (Figure 9F). This immunoprofile supported smooth muscle differentiation and helped exclude a GIST or other mimics in this context. The Ki-67 labelling index of approximately 20% was seen in hot spots (Figure 10).

**Figure 9:**
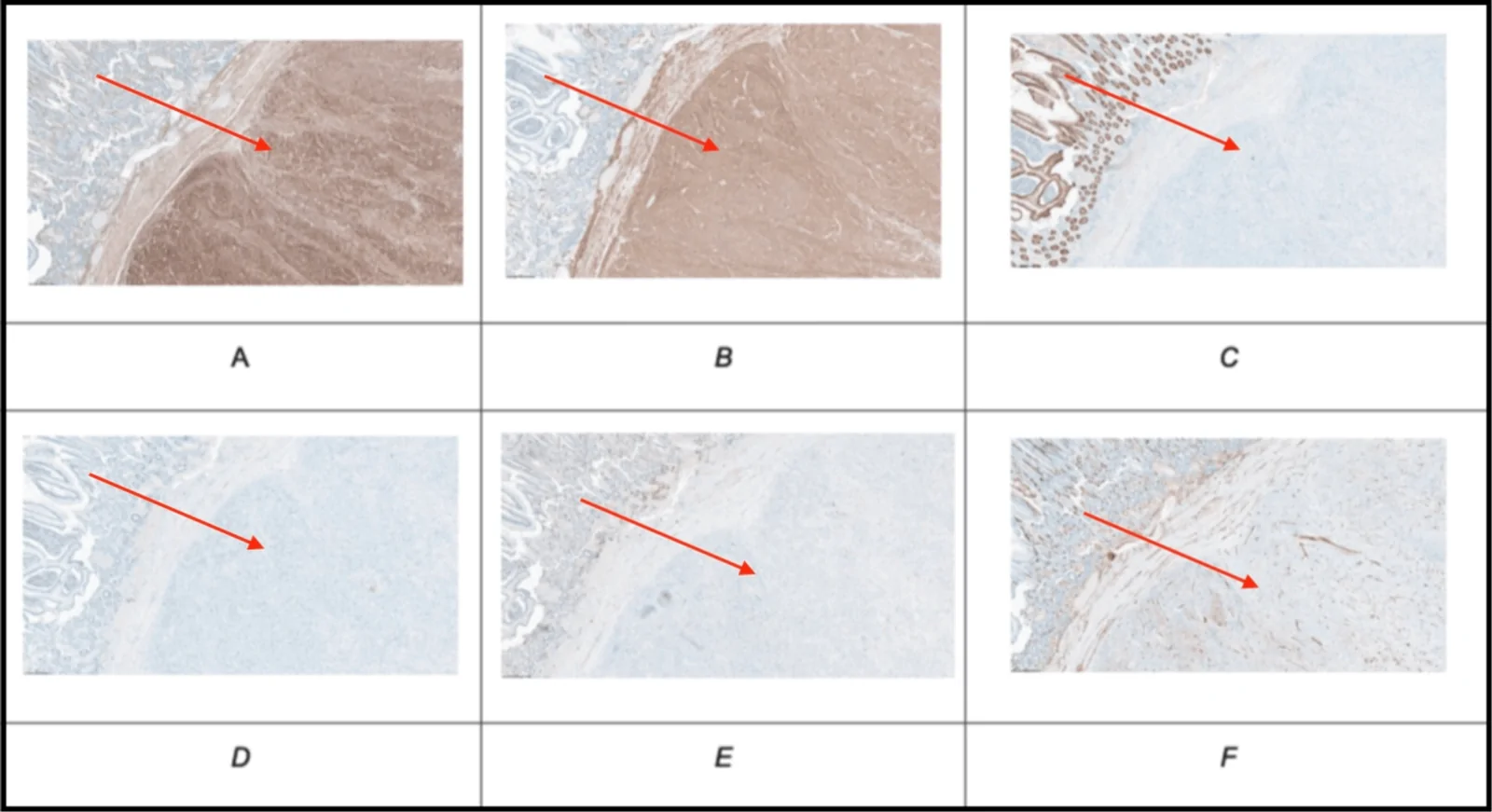
Immunohistochemistry findings that support smooth muscle cell differentiation (×5 magnification). The tumour shows diffuse strong positivity for smooth muscle actin (A) and desmin (B), with no reactivity for AE1/3 (C), S-100 (D), CD117/c-kit (E) and CD34 (F). All indicated by red arrows.

**Figure 10:**
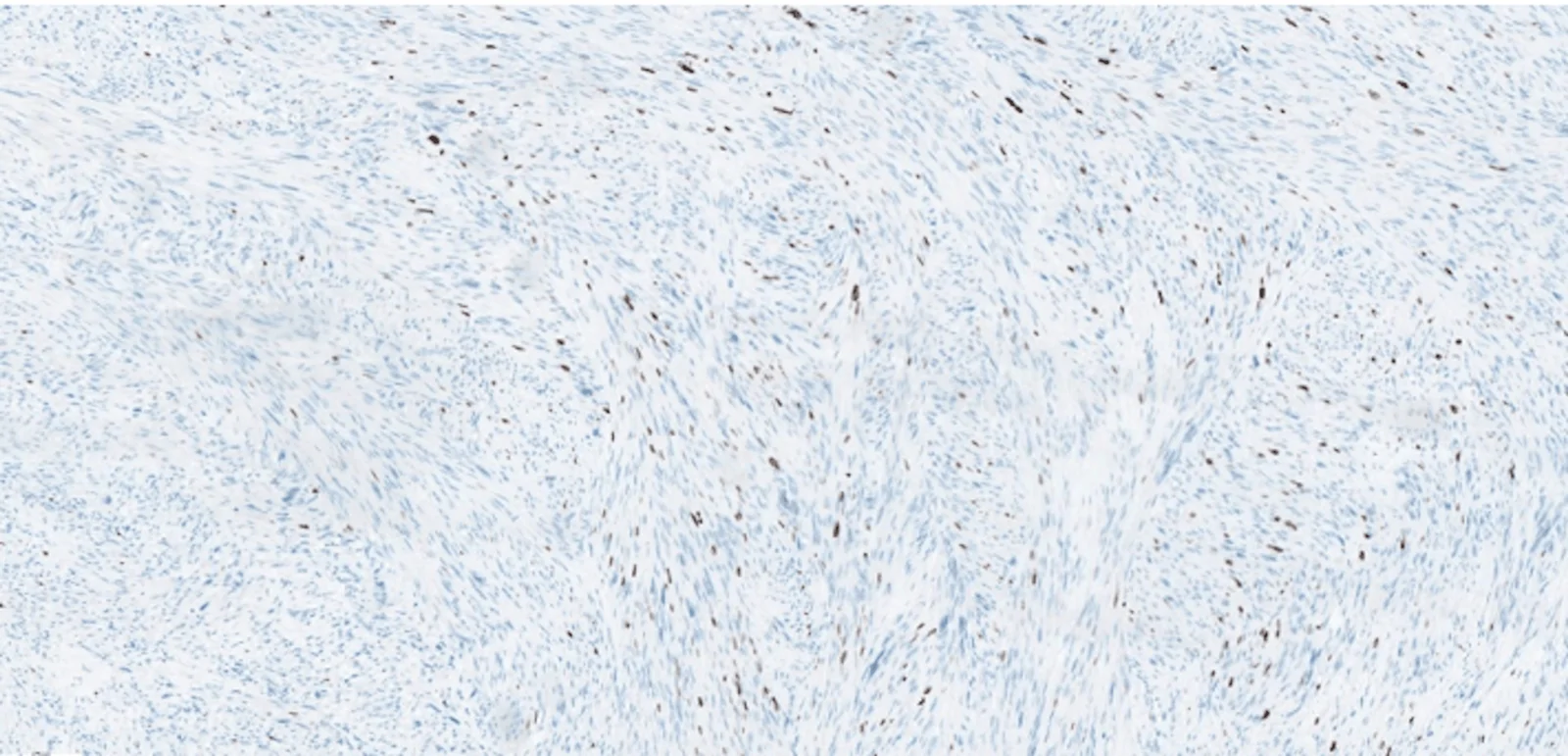
Ki-67 proliferation index of approximately 20% in hotspots at ×10 magnification.

The combination of gross morphology, histological features of spindle cell fascicles with cellular atypia, high mitotic activity, infiltrative growth limited to the bowel wall, and a smooth muscle immunophenotype supported the diagnosis of small bowel LMS. All resection margins were clear, and no lymph node metastases were identified.

Computed tomography of the chest was performed 10 days postoperatively to assess for metastatic disease and was unremarkable, with no solid lung nodules or mass lesions identified. The patient was asymptomatic during his one-month follow-up clinic appointment. The case was further discussed at the sarcoma multidisciplinary team meeting. Another computed tomography of the chest, abdomen, and pelvis was performed on postoperative day 47, and there was no evidence of disease recurrence. At the two-month follow-up, his anaemia had resolved, his full blood count was within normal limits, and his haemoglobin level rose from 107 g/L before surgery to 133 g/L. He had received no perioperative blood transfusions. Iron studies showed a serum iron level of 9 µmol/L, a transferrin level of 2.7 g/L, and a transferrin saturation of 13%. These studies suggested mild residual iron deficiency, although a ferritin level was not available and the contribution of his iron supplementation could not be determined. Table 2 compares iron studies performed one month before surgery and two months after surgery. His liver function tests and renal function tests were also normal. The patient was advised to attend three-monthly follow-up appointments with repeat imaging.

**Table 2:** Comparison of iron studies one month before and two months after surgery. Reference ranges are based on the local clinical biochemistry user guide.

	1 month before surgery	2 months after surgery	Reference range (male)
Serum iron (µmol/L)	4.7	9.0	11–28
Transferrin (g/L)	2.06	2.70	2.00–3.60
Transferrin saturation (%)	9	13	20–50

## Discussion

Adult intussusception accounts for approximately 5% of all intussusception cases, with the majority occurring in the paediatric population. It represents 1% of all small bowel obstructions and is reported in fewer than 1 in 1,300 abdominal operations [2]. In contrast to paediatric patients, adult cases often have an underlying pathological LP. Neoplasms, either benign or malignant, account for 60% of identified LPs [2]. Other non-neoplastic, non-idiopathic causes include postoperative adherences, coeliac disease, Crohn’s disease, endometriosis, and infections [2]. Based on anatomical location, intussusception can be classified into the following four categories: (i) entero-enteric (involves the small bowel only), (ii) ileo-cecal (the ileocecal valve serves as the LP for intussusception), (iii) ileo-colic (the terminal ileum telescopes into the ascending colon), and (iv) colo-colic (involves the large bowel only) [3,5]. Ileo-ileal intussusception is a specific subtype within the entero-enteric category [3]. Adult intussusception may present with abdominal pain, nausea, vomiting, and a palpable abdominal mass on physical examination [1,2]. Other associated symptoms such as weight loss may indicate the presence of an underlying serious pathology, including a malignant LP [6]. If left untreated, necrosis, peritonitis, sepsis, and perforation may occur [2].

Computed tomography is the imaging modality of choice for diagnosing adult intussusception. Typical computed tomography findings include the ‘target’ or ‘sausage’ sign and ‘bowel-within-bowel’ appearance [1]. Surgery is the recommended, definitive treatment for adult intussusception [1]. The surgery can be performed with or without a primary reduction, although reduction before resection may allow a more limited bowel resection [1]. However, the risks of reduction before resection include intraluminal seeding and venous embolisation of malignant cells in the region of the ulcerated mucosa, bowel perforation, and anastomotic complications when the bowel is oedematous and inflamed [1].

LMS is a malignant soft-tissue neoplasm of smooth muscle differentiation that may arise in various organ systems, including the bladder, gastrointestinal tract, uterus, or blood vessels. Small bowel malignancies account for approximately 2% of all gastrointestinal malignancies [4]. A population-based study showed that adenocarcinomas accounted for 38% of all small bowel malignancies, followed by neuroendocrine tumours (35%), lymphomas (15%), and sarcomas (12%) [7]. Small bowel LMS is a subtype of sarcoma, accounting for approximately 0.1% to 3% of small bowel malignancies [4]. Patients with small bowel LMS may present with chronic abdominal pain, weight loss, gastrointestinal bleeding, and anaemia [4].

Diagnosing small bowel LMS involves imaging techniques, histopathology, and immunohistochemistry. On computed tomography, these cancers are heterogeneous, show low central attenuation due to necrosis, and may show calcification, although this is exceedingly rare [8]. On magnetic resonance imaging, these tumours often demonstrate cystic foci, with signal intensity varying according to the imaging sequence [8]. On T1-weighted imaging, they are isointense to muscle, while on T2-weighted imaging, they are intermediate to hypointense relative to neighbouring fat tissue [8]. On gross pathology, they appear as white-grey masses and, when large, they may be associated with haemorrhage, necrosis, and cystic changes [9]. Microscopically, they display long intersecting fascicles of spindle-shaped cells with nuclear pleomorphism [9]. LMS may histologically resemble GIST, as both may present as spindle cell malignant neoplasms with high mitotic counts, necrosis, and cytological atypia. Immunohistochemistry is essential to differentiate these tumours. In small bowel LMS, positive staining for smooth muscle markers such as SMA and desmin supports the diagnosis, while negative staining for CD117 helps distinguish it from GIST [9]. In our patient, the tumour matched the macroscopic, microscopic, and immunohistochemical features of a typical small bowel LMS. Treatment of small bowel LMS primarily involves complete surgical resection of the tumour with negative margins [4].

Similar cases of small bowel LMS causing intussusception have previously been reported [10-13]. Table 3 compares this case with previously reported cases. Štor et al. [10] reported that before admission, their patient had undergone oesophagogastroduodenoscopy and colonoscopy due to abdominal pain and anaemia. These demonstrated erosive gastritis and sigmoid colon diverticulosis, respectively, which may have provided alternative explanations for the anaemia. Rayasam et al. [13] also reported that upper gastrointestinal endoscopy performed one month before surgery was normal. In contrast, the repeated upper and lower endoscopic investigations in our patient were unremarkable and failed to identify a source for the IDA. At the two-month follow-up after resection of the LMS, the anaemia resolved, although iron studies showed mild residual iron deficiency. The histological finding of tumour-associated mucosal ulceration provides a plausible source for chronic occult gastrointestinal blood loss that may have contributed to the patient’s long-standing unexplained IDA. Among the other published cases reviewed, similar repeated gastrointestinal investigations to identify the source of IDA before presentation were not described.

**Table 3:** Comparison of previously reported cases of small bowel leiomyosarcoma presenting with intussusception.

Case report	Age and sex	Intussusception site	Clinical presentation	Relevant medical history	Relevant imaging findings	Treatment	Pathological findings	Follow-up
Štor et al. (2019) [10]	80, male	Ileo-ileal	Abdominal pain, constipation	Anaemia, arterial hypertension, dyslipidaemia	X-ray of the abdomen showed air-fluid levels of the small intestine. Ultrasonography of the abdomen confirmed intussusception of the small intestine. CT scan showed ileo-ileal intussusception with the ‘target’ sign and dilated small bowel loops	Exploratory laparotomy with resection of the affected ileal segment and primary entero-enteral anastomosis	4.8 cm leiomyosarcoma at the terminal ileum. The tumour involved the full thickness of the ileum. One adjacent lymph node was retrieved, with no evidence of metastatic involvement. Immunohistochemistry profile: CD117-, DOG1-, desmin+, caldesmon+. Ki-67 index 60%. Presence/absence of mucosal ulceration not reported	No adjuvant therapy. The patient remained without evidence of disease for 12 months after the initial presentation
Mazzotta et al. (2020) [11]	90, male	Ileo-ileal	Abdominal pain, nausea, bowel occlusion	Anaemia, hypertension, dyslipidaemia, right open inguinal hernia repair, haemorrhoidectomy	X-ray of the abdomen showed air-fluid levels without subdiaphragmatic free gas. CT scan demonstrated the classic ‘bulls-eye’ appearance, suggesting intussusception	Exploratory laparotomy with ileocecal resection and latero-lateral isoperistaltic ileocolic anastomosis	The tumour was on the muscular layer of the small bowel. Immunohistochemistry profile: actin+, desmin+, CD117-, CD34-, DOG1-, S100-. Diagnosis of leiomyosarcoma G2 grade FNCLCC. Presence/absence of mucosal ulceration not reported	After oncological evaluation, no further surgery or medical treatment was needed
Makwana et al. (2025) [12]	58, male	Ileo-ileal	Abdominal pain, vomiting, postprandial fullness, constipation, significant weight loss with anorexia over 1 month	Hypertension for 9 months	X-ray of the abdomen suggested air- and fluid-filled bowel loops. Ultrasonography suggested ‘bowel-within-bowel’ appearance and ‘target’ sign. Contrast-enhanced CT showed ‘bowel-within-bowel’ appearance suggesting intussusception, with no evident lead point identified	Exploratory laparotomy with ileal resection and ileo-ileal anastomosis	Histopathology suggestive of high-grade leiomyosarcoma. Mucosa involved but no lymphovascular invasion. Pathological staging: T1N0MX. Immunohistochemistry profile: desmin+, CD117-, DOG1-, ALK-. Ki-67 index 30%. Presence/absence of mucosal ulceration not reported	Uneventful postoperative period. Oncology suggested no active intervention and further follow-up
Rayasam et al. (2026) [13]	60, sex not reported	Ileo-ileal	Recurrent abdominal pain	Iron-deficiency anaemia	Contrast-enhanced CT showed ileo-ileal intussusception with homogeneously enhancing intraluminal mass. MRI demonstrated a polypoid intraluminal mass with relatively low T2 signal intensity, post-contrast enhancement and diffusion restriction. Axial fat-suppressed post-contrast T1-weighted image showed the classic ‘target’ sign	Laparoscopic exploration with segmental small bowel resection with stapled side-to-side anastomosis	Histopathology showed spindle cell neoplasm with nuclear atypia and frequent mitoses. Immunohistochemistry profile: SMA+, CD117-, DOG1-, S100-, ALK1-. Ki-67 index 40%. Presence/absence of mucosal ulceration not reported	Surveillance imaging showed no evidence of local recurrence or metastatic disease. Ongoing surveillance
Present case (2026)	60s, male	Ileo-ileal	Abdominal pain, constipation, nausea, vomiting, unintentional weight loss of 6.4 kg in a 10-day period	Iron-deficiency anaemia	CT of the abdomen and pelvis showed ‘target’ sign and ‘bowel-within-bowel’ appearance consistent with intussusception. On axial view, upstream small bowel dilatation was noted, suggesting small bowel obstruction	Laparoscopic-assisted segmental small bowel resection and side-to-side anastomosis	Histopathology and immunohistochemistry confirmed leiomyosarcoma. Mucosal ulceration was present. Resection margins were clear, and no lymph node metastases were identified	Early surveillance imaging showed no evidence of local recurrence or metastatic disease. Ongoing surveillance was planned. At the two-month follow-up, the anaemia had resolved with mild residual iron deficiency

## Conclusions

Adult intussusception requires early recognition because the presentation is often nonspecific, and delayed management can lead to severe complications such as bowel ischaemia, necrosis, and perforation. It is also important to consider an underlying malignant LP, especially when associated features such as unintentional weight loss and IDA are present. In this case, tumour-associated mucosal ulceration provided a plausible source of chronic occult gastrointestinal blood loss that may have contributed to the patient’s long-standing unexplained IDA. This case highlights small bowel LMS as a rare malignant LP for ileo-ileal intussusception. Timely imaging and prompt surgical intervention enabled definitive treatment. Immunohistochemistry was vital in confirming the diagnosis of small bowel LMS and excluding histological mimics, particularly GIST. Continued surveillance and multidisciplinary follow-up are important to monitor for disease recurrence or metastatic disease.
